# Prenatal environment impacts telomere length in newborn dairy heifers

**DOI:** 10.1038/s41598-023-31943-8

**Published:** 2023-03-22

**Authors:** Maya Meesters, Mieke Van Eetvelde, Dries S. Martens, Tim S. Nawrot, Manon Dewulf, Jan Govaere, Geert Opsomer

**Affiliations:** 1 Department of Internal Medicine, Reproduction and Population Medicine, Faculty of Veterinary Medicine, Ghent University, Merelbeke, Belgium; 2 Centre for Environmental Sciences, Hasselt University, Diepenbeek, Belgium; 3 Research Unit Environment and Health, Department of Public Health & Primary Care, Leuven University, Leuven, Belgium

**Keywords:** Animal physiology, Ageing

## Abstract

Telomere length is associated with longevity and survival in multiple species. In human population-based studies, multiple prenatal factors have been described to be associated with a newborn’s telomere length. In the present study, we measured relative leukocyte telomere length in 210 Holstein Friesian heifers, within the first ten days of life. The dam’s age, parity, and milk production parameters, as well as environmental factors during gestation were assessed for their potential effect on telomere length. We found that for both primi- and multiparous dams, the telomere length was 1.16% shorter for each day increase in the calf’s age at sampling (P = 0.017). The dam’s age at parturition (P = 0.045), and the median temperature-humidity index (THI) during the third trimester of gestation (P = 0.006) were also negatively associated with the calves’ TL. Investigating multiparous dams separately, only the calf’s age at sampling was significantly and negatively associated with the calves’ TL (P = 0.025). Results of the present study support the hypothesis that in cattle, early life telomere length is influenced by prenatal factors. Furthermore, the results suggest that selecting heifers born in winter out of young dams might contribute to increased longevity in dairy cattle.

## Introduction

In Holstein Friesian (HF) cows, high milk yield has been associated with a reduced longevity^1^. As modern dairy cows are criticised for their short productive lifespan and their adverse environmental footprint, longevity is currently encouraged by public opinion^1,2^. For the dairy industry, longevity is an economically important trait resulting in reduced culling rates, and thus in a lower need of replacement heifers^3^.

In multiple species including humans^4^, cattle^5^, sheep^6^, birds^7^, and badgers^8^, longevity has been linked to leukocyte telomere length. In humans, telomere length (TL) is considered to be a biological marker of aging^9^. Telomeres are nucleoprotein structures found at the ends of linear eukaryotic chromosomes, consisting of TTAGGG repeats^10,11^. They protect chromosomal integrity, inhibit aberrant fusions and rearrangements occurring on broken chromosomes and are crucial for the complete replication of genomic DNA^11^. Telomeres shorten with every cell division^10,11^ and in response to inflammation and oxidative stress^12–14^, but can be maintained through telomerase activity^10,14^. When TL declines to a critical threshold, cellular replicative senescence or cell death is induced^15,16^. As such, telomere dynamics are closely linked to cell function and cellular aging, and also seem to be associated with organismal aging^12,17^.

In human population-based studies, TL has been associated with an individual’s lifespan and disease risk^18,19^, as it is described to be a predictor of disease onset for cardiovascular disease, diabetes, and mortality risk^20–22^. Similar to what has been described in humans, longevity or productive lifespan^23,24^, as well as a cows’ future health status^25^ have been associated with leukocyte telomere length (LTL) and telomere attrition rate in dairy cattle^23–25^.

Factors influencing TL in humans, such as sex^26^, oxidative stress^27^, genetics^28^, and acquired disease^29^, are well described. Sex^30^ and breeding status^16^ have been described to influence LTL in sheep, whilst in cattle, age and herd^5^, genetics^23^, and high ambient temperatures^24^ have been shown to exert an influence.

Human TL at birth shows a high variability, comparable with the age-adjusted TL variability in adults^31^. Addressing gene-environmental factors that contribute to an individual’s initial TL may provide important insight in the developmental origins of health and disease conditions related to TL. This is in line with ‘The fetal programming of telomere biology hypothesis’, which states that TL and telomerase expression may be programmed by maternal states and stress conditions during pregnancy in women^32^.

Developmental programming, also referred to as prenatal or fetal programming, refers to the concept that adverse conditions in utero or during infancy can increase the risk of disease and premature aging in later postnatal life^33–36^. Prenatal programming has been well described in both humans^37,38^ and livestock^33,39–42^.

Prenatal programming of TL is an interesting observation, as TL can be tracked throughout time and the ranking of TL appears to be stable over time, implying that the initial setting of newborn TL contributes significantly to TL later in life^43–45^. In humans, prenatal maternal stress^46^, maternal pre-pregnancy BMI^9^, paternal age^47^, maternal pro-inflammatory state^48^, ambient temperatures during pregnancy^49^ and maternal vitamin D intake^50^, amongst others, are reported to influence newborn TL. A birth cohort effect, which may reflect environmental factors, has been described in sheep^6^ and badgers^8^, whilst lameness during pregnancy has been shown to shorten TL in newborn dairy calves^25^. To the best of our knowledge, prenatal programming of TL has only been scarcely described in non-human species.

Previous work by our research group has shown that prenatal factors contribute in determining a cow’s intrinsic ability to achieve a long and productive life^51^. Identifying a biological indicator to confirm this research would enable farmers to use this underlying biological mechanism to select animals with a predicted longer life to increase longevity of their livestock.

Therefore, the aim of the present study was to identify prenatal factors that are associated with LTL in newborn dairy heifers. We hypothesised that maternal age and parity, parameters associated with milk yield, and heat stress during gestation could be important in programming the early-life LTL in dairy cattle.

## Results

### Calf, dam and TL characteristics

The 210 calves included in this study were all purebred singleton female HF calves, which were born at four Flemish (Belgium) herds, between the summer of 2017 and the fall of 2018. Blood sampling was performed within 10 days after birth.

The general characteristics of the studied population (n = 210) are provided in Table 1. A detailed distribution of these characteristics within each herd can be found in the Supplementary file (S1). The calves had a mean [± standard deviation (SD)] gestational age of 278 ± 4.7 days and most were born during the fall months (44.8%). Their mean age at sampling was 4 ± 2.2 days, and the mean bodyweight at sampling was 40.5 ± 4.72 kg. The mean dam age at parturition was 39.9 ± 18.3 months, and more than half of the dams was multiparous (58%). The mean milk yield during gestation (from conception until drying off) was 7.105 ± 1.627.3 kg, of which 3.094 ± 685.2 kg was produced during the first trimester of gestation.

**Table 1:** Calf and dam characteristics of the studied population.

Characteristic	Mean ± SD or n (%)
Calves	
Number of calves per herd*	
1	46 (21.9)
2	82 (39.0)
3	44 (21.0)
4	38 (18.1)
Birth season	
Spring	20 (9.5)
Summer	68 (32.4)
Fall	94 (44.8)
Winter	28 (13.3)
Gestation length (days)	278 ± 4.7
Age at sampling (days)	4 ± 2.2
Body weight at sampling (kg)	40.5 ± 4.72
Heart girth at sampling (cm)	79.1 ± 3.21
Withers height at sampling (cm)	76.1 ± 2.90
Diagonal length at sampling (cm)	72.4 ± 3.27
Dams	
Dam age at parturition (months)	39.9 ± 18.3
Age primiparous	23.4 ± 1.8
Age multiparous	50.0 ± 16.7
Parity^a^ (range)	1 to 9
Primiparous dams	88 (42)
Multiparous dams	122 (58)
Calving interval (days)	379 ± 60.2
Length of the dry period (days)	44 ± 12.0
Milk yield during gestation (kg)	7.105 ± 1627.3
During first trimester	3.094 ± 685.2
During second trimester	2.605 ± 597.2
During third trimester	1.417 ± 438.1

^a^Parity 1 = after the first parturition.

*A detailed distribution of all characteristics across herds is given in Supplementary file S1.

The calves’ TL was on average 1.01 ± 0.17, ranging from 0.66 to 1.66. The median of the TL was 0.99 and the 25th and 75th percentile were 0.90 and 1.12, respectively. Distributions of the untransformed and log_10_-transformed TL are given in Supplementary file S2.

### Univariable analysis

In the univariable analysis (Table 2), 23 of the evaluated variables showed an association with the log_10_ transformed TL (P < 0.15). These variables included those related to the calf: season of conception (P = 0.074), month of conception (P = 0.018), season of birth (P = 0.070), month of birth (P = 0.002), calf age at sampling (P = 0.013), and calf heart girth (P = 0.038).

**Table 2:** Results of the univariable model showing variables associated with the log_10_ transformed TL (P < 0.15).

Variables	Categories	Estimates^a^	P-value
Calf			
Season of conception	Spring	Ref.	0.074
	Summer	− 0.842	
	Fall	− 7.069	
	Winter	− 7.218	
Month of conception			0.018
Season of birth	Spring	Ref.	0.070
	Summer	− 6.816	
	Fall	− 7.490	
	Winter	− 0.895	
Month of birth			0.002
Calf age at sampling		− 1.233	0.013
Calf heart girth		− 0.708	0.038
Dam			
Dam parity (continuous)		− 1.457	0.062
Dam parity (categorical)	1	Ref.	0.133
	2	− 0.618	
	≥3	− 4.755	
Dam age at calving (months)		− 0.122	0.045
MilkBot^**®**^ scale		− 0.200	0.121
MilkBot^**®**^ milk at peak		− 0.276	0.129
MilkBot^**®**^ milk at 60 DIM		− 0.005	0.121
THI			
Mean THI first trimester		0.455	< 0.001
Days THI > 65 first trimester		0.137	0.002
Days THI > 70 first trimester		0.221	0.004
Mean THI third trimester		− 0.414	0.002
Days THI > 65 third trimester		− 0.133	0.002
Days THI > 70 third trimester		− 0.112	0.003
Median weekly THI		− 0.436	0.054
Median weekly THI first trimester		0.451	< 0.0017
Median weekly THI third trimester		− 0.388	0.003
Weekly THI’s week 25 until week 38^b^			Between <0.001 and 0.071
Days THI > 70 (whole gestation)		− 0.126	0.020

^a^Estimates presented as a % difference in TL for a 1-unit increase in the explanatory variable.

^b^Detailed weekly THI’s between week 20 and 39, see Supplementary file S3.

Dam related variables that were tested in the univariable analysis were: dam parity (continuous, P= 0.062), dam parity (categorical, P = 0.133), dam age at calving (P = 0.045), MilkBot^®^ scale (P = 0.121), MilkBot^®^ peak (P = 0.129), and MilkBot^®^ 60 DIM (P = 0.121).

Finally, the THI variables selected in the univariable analysis were: mean THI first trimester (P < 0.001), days THI > 65 first trimester (P = 0.002), days THI > 70 first trimester (P = 0.004), mean THI third trimester (P = 0.002), days THI > 65 third trimester (P = 0.002), days THI > 70 third trimester (P = 0.003), median weekly THI (P = 0.054), median weekly THI first trimester (P < 0.001), median weekly THI third trimester (P = 0.003), weekly THI’s between week 25 until week 38 (between resp. P < 0.001 and P = 0.071), and lastly days THI > 70 during the whole gestation (P = 0.020).

### Multivariable analysis

The results of the multivariable model with the best fit for all animals (primi- and multiparous) and for the multiparous animals alone are shown in Tables 3 and 4, respectively. The R^2^ for both models revealed that a limited proportion of variance is explained by the variables in the model. The R^2^ for the model including all animals was 19%, of which 8% was explained by the fixed factors in the model. The R^2^ for the model for multiparous dams was 21%, of which 9% was explained by the fixed factors. Hence, in both models, 11% of the variation in TL is explained by the random factor, herd.

**Table 3:** Multivariable model (both primi- and multiparous dams included) adjusted from the variables that obtained P < 0.15 in the univariable analyses.

Fixed effect	Estimate (95% CI)^a^	P-value
Calf age at sampling (days)	− 1.16 (− 2.089, − 0.205)	0.017
Dam age at calving (months)	− 0.12 (− 0.233, − 0.002)	0.045
Median THI third trimester of gestation	− 0.35 (− 0.597, − 0.104)	0.006

^a^Estimates, with 95% confidence interval, presented as a % difference in TL for a 1-unit increase in the explanatory variable.

Herd was included as a random factor and the log_10_ transformed TL was used as the outcome variable.

**Table 4:** Multivariable model for the multiparous dams (n=122), adjusted from the variables that obtained P < 0.15 in the univariable analyses.

Fixed effect	Estimate (95% CI)^a^	P-value
Calf age at sampling (days)	− 1.57 (− 2.882, − 0.190)	0.025
MilkBot^**®**^ scale (kg/day)	− 0.22 (− 0.465, − 0.022)	0.078
Days THI >65 during third trimester of gestation	− 0.08 (− 0.188, -0.012)	0.098

^a^Estimates, with 95% confidence interval, presented as a % difference in TL for a 1-unit increase in the explanatory variable.

Herd was included as a random factor and the log_10_ transformed TL was used as the outcome variable.

Calf age at sampling, dam age at calving and median THI during the third trimester of gestation were significantly and negatively associated with TL in newborn calves. Cows that were older at parturition birthed calves with shorter TL (P = 0.045), and a higher median THI during the third trimester of gestation resulted in calves born with shorter TL (P = 0.006). However, the largest effect was seen in calf age at sampling, with significantly shorter TL (P = 0.017) in calves that were older at sampling.

For the model including multiparous dams, only calf age at sampling was significantly and negatively associated with the newborns TL. The MilkBot^®^ scale, and the days with a THI >65 during the third trimester of gestation tended to be negatively associated with the TL of newborn calves. Calves that were older at sampling had significantly shorter TL (P = 0.025). The higher the MilkBot® scale of the dam, the shorter the TL of newborn calves tended to be (P = 0.078), and more days with a THI >65 during the third trimester of pregnancy, tended (P = 0.098) to amount to shorter TL in newborn calves.

## Discussion

Telomere length at birth has a significant association with survival, length of productive life, and the future health status in cattle^25^. Thus, in the context of developmental programming of health and disease, it is important to explore parental and environmental factors that are associated with TL at birth.

To the best of our knowledge, this study is the first to examine prenatal programming of bovine telomere biology. We found that for primi- and multiparous dams, calf age at sampling, dam age at calving and median THI during the third trimester of gestation were associated with shorter TL at birth. Looking at the multiparous animals separately, only calf age at sampling showed a significant effect on TL of the newborn calves. Our findings cast a light on prenatal influences on TL, which may contribute to longevity after birth.

There is a significant, negative effect of calf age at sampling on the TL in both of our models. This is in accordance with the study of Seeker et al. (2019) that describes a clear decline in LTL in the first year of life^23^. A faster telomere attrition rate shortly after birth has not only been described in cattle^23^, but also in humans^52^ and Soay sheep^6^. It has been proposed that faster attrition shortly after birth is due to the high number of cell divisions necessary for quick growth. Furthermore, postnatal maturation of the immune system and sudden pathogenic challenges might cause fast telomere depletion during the first months of life^23^. A point of improvement would be to sample calves within the first 24 hours of birth, to have minimal variation in age at sampling as well as to minimise the telomere attrition happening shortly after birth.

In our study, TL differed significantly between herds. A similar variation in TL has been described in previous research and could be attributed to herd environment^5^, which may be due to differences in genetics and management. As our samples were placed on the qPCR plates sorted by herd, plate and herd effects might reflect the same thing. However, we technically adjusted qPCR plate effects by including inter-run calibrators. Including both effects in the statistical models would proclaim too much weight on one or both, thus, it was decided to include only herd as a random effect.

Another finding was that for all animals (both primi- and multiparous), TL was negatively associated with dam age at parturition, in other words, the older the dam at calving, the shorter the calf’s TL. In a separate analysis including only multiparous animals, no such effect was observed, suggesting that this effect comes mainly from the primiparous animals. One might speculate that this might be due to the fact that during pregnancy, these adolescent dams are still growing substantially themselves as opposed to multiparous animals^53^. However, this finding is in contrast to what has been described in the great reed warbler, where maternal age was strongly and positively correlated with TL^54^. Conversely, no parental age effects on offspring TL where found in free-living Soay sheep and badgers^14,55^. González-Recio et al. clearly established that being born out of heifers leads to offspring with a longer functional lifespan, compared to animals born out of multiparous dams^56^. Also, previous research by our group found similar results, comparing dam parity with the odds of the calf producing at least 100,000 kg of milk during its productive life. Producing 100 tonnes of milk is an achievement that is indicative of longevity combined with a high lifetime production. It was shown that the odds of becoming a 100 tonne cow was highest in cows born out of heifers (parity 1 vs. parity 2, OR = 1.58), in other words, born out of young animals^51^. Consequently, it might be interesting to improve longevity by selecting replacement heifers born out of younger dams.

Interestingly, none of the milk yield parameters were associated with TL in the multivariable model for all animals or multiparous dams separately. Neither dry period length nor total milk yield during gestation were significantly associated with TL. MilkBot^®^-parameters milk yield at peak production and milk yield at 60 DIM were associated with TL in the univariable analyses, but not in the multivariable models. In multiparous animals, only the MilkBot^®^ scale showed a weak negative association with the TL of newly born calves. The MilkBot^®^ scale signifies the overall magnitude of the milk production, and is the theoretical maximum daily milk yield (lb/day or kg/day). It rises with parity and varies between breeds, with Holstein Friesians having the greatest scale values^57^. Seeker et al.^23^ proposed in their study that two genetic groups considerably different in milk yield, did not differ in their mean TL, suggesting it is likely that there is no unfavourable genetic correlation between TL and productivity^23^. This would be desirable, as it implies that selection for longevity, based on TL, would have no effect on milk production of cows. However, our results show there is a tendency to shorter telomeres in newborn calves born out of dams with a larger scale according to the MilkBot^®^ model. This could imply that the magnitude of the milk production of the dam might influence the calves’ TL negatively, thus have an impact its possible longevity. This tendency might be more pronounced when more multiparous dams, significantly differing in genetic potential for milk production, would be included in the study, since the multiparous dams in our study had similar milk yields. Further research in a larger number of lactating animals might elucidate an effect of milk yield parameters on TL at birth. However, the global population of Holstein Friesians can be considered as one single population unit in terms of genetic divergence^58^, thus these effects might only be demonstrated at the extremes of the population.

Other than human studies, this is the first study investigating the influence of heat stress during pregnancy on TL in newborn animals. Seeker et al.^24^ hypothesised that the year of sampling was associated with telomere attrition rates, and that these changes in TL might be partially explained by weather variables. This led us to investigate these weather variables more deeply by taking into account the maximum daily temperatures and daily relative humidity during the entire gestation of the dam. We found that the median THI during the third trimester of gestation was negatively associated with the calves’ TL. Such that every one-unit increase of the median third trimester THI, lead to a 0.35% decrease in TL. In the multiparous dams alone, there was a tendency that during the third trimester of gestation, the amount of days with a THI above 65 was negatively associated with the TL at birth. These results are in agreement with what has been described recently in humans, by Martens et al.^49^. They describe a clear negative effect of prenatal temperature exposure above the heat threshold (19.5 °C) on the TL of umbilical cord blood in newborn babies. The effect of a 1 °C increase in ambient temperature was strongest at week 36 of pregnancy, and led to a 3.29% decrease of the cord TL at this timepoint. This study also described an interesting protective effect of cold temperatures on TL, with the cold threshold set at 5 °C^49^. The protective effect of cold exposure may be due to lower metabolic rates and altered oxidative stress states^59^. Additionally, an immune-stimulating effect of acute cold exposure has been described in humans^60^. Because of this protective effect, one might speculate that ventilation and/or cooling measures during periods of heat stress might reduce the loss of TL in newborn calves. The potential protective effect of cold temperatures or other factors related to seasonality, remain to be investigated.

A limitation in our research might be that the health status of the dam during pregnancy was unknown. It has been previously described that lameness status of the dam during gestation had a significant effect on TL at birth, with calves of lame cows having shorter TL at birth^25^. In humans, it has recently been shown that a maternal pro-inflammatory state during pregnancy was significantly associated with shorter TL in newborn babies^48^. Thus, the effects of lameness or other disease events during gestation could be interesting to investigate further. The absence of paternal data might be another shortcoming in our study. Although sire identification was available for all calves, the use of sires showed great variance both within as well as between herds. As such, sire effects were considered included in the herd effects. Also, sire age at semen collection was impossible to ascertain, thus further investigation of paternal age was not possible. Strong paternal effects have been described in humans, in a study by Nordfjäll et al*.*, where paternal age was positively correlated with the newborn’s TL^47^. This positive paternal age effect was not demonstrated in other species like sheep and badgers^14,55^, although investigating paternal data further could be worthwhile.

It is important to note that the R^2^ of our statistical models is low. In our study, only about 20% of the variance in TL is explained by the variables in our models, of which 11% can be explained by herd. Thus, herd-related factors (e.g. management and genetics) might be considered as important influences on TL variance. Accordingly, about 80% of the variance in TL cannot be explained by the variables in our models but might largely be due to inheritance. Heritability of TL has been described to be moderate (44.9%) across vertebrate species, although there is considerable heterogeneity in heritability estimates between these species^61^. In sheep heritability was estimated to be 23.3%^13^, whereas in dairy cattle heritability was described to be 36% at birth to 46% at first lactation^25^. In humans, heritability estimates have been shown to be in the order of 60%, although lower and higher estimates have been demonstrated^62^. Further studies are needed to investigate other prenatal effects on TL in newborn calves. Also, more longitudinal studies are needed to assess these prenatal effects on the newborn’s TL, and the consequences later in life. While heat stress is a hot topic in modern dairy cattle research, its effects on TL as well as the effects of preventative measures warrant further investigation.

## Materials and methods

### Animal population and data collection

Animals included in the present study were Holstein Friesian dairy cows and their respective calves, belonging to 4 dairy farms in Flanders (Belgium). Herds were selected based on their willingness to collaborate and the availability of necessary data. Informed consent was obtained from all dairy farmers. Herd sizes ranged from 100 to 250 lactating cows, with an average 305-days milk yield between ~9000 and ~11,000 kg. All herds participated in official milk recording. In three herds, cows were milked twice a day while in the fourth herd cows were milked by an automated milking system, which recorded an average of 2.6 milkings a day.

Animals were housed in free-stall barns and were fed according to their requirements for maintenance and production, based on results of the monthly production tests. Rations consisted of high-quality roughages (maize and grass silage, sugar beet pulp, and fodder beets), supplemented with concentrates. Cows were dried off between six to eight weeks prior to their expected calving date. When the animals approached parturition, they were separated in a maternity pen and closely monitored by the farmer. After calving, all calves were immediately moved to individual calf pens with straw bedding and were given 2L colostrum. Care was taken that all calves received 4 L of colostrum within 12 hours after birth.

All purebred singleton female HF calves born at the participating herds between August 2017 and November 2018, were enrolled in the study. Newborn calves were weighed (Seca^®^ flat scale, Seca Benelux, Naarden, the Netherlands), and blood samples were taken within 10 days after birth. Apart from blood sampling and weighing, body measurements of the calves were measured including heart girth, withers height, and diagonal length, as described by Kamal et al*.*^63^. Season of birth was grouped as follows: winter (21 December to 20 March), spring (21 March to 20 June), summer (21 June to 20 September), and fall (21 September to 20 December).

Data selection comprised of correct identification of the calves, gestation length, sex, and age at blood sampling. Only calves born after a gestation length of between 265 and 295 days were included in the study. Blood samples had to be taken within ten days after birth. As such, data of 210 calves were included for further analyses.

Prenatal environment was interpreted both in the narrow sense of the word (as uterine environment, thus maternal factors), as well as in the broader sense, meaning climatic conditions during the whole gestation.

Dam information was extracted from the herd databases and included parity, dam age, and monthly milk production via official milk recording. Monthly milk records of the dams were fitted to the MilkBot^®^ model to summarize the magnitude and shape of each lactation curve. MilkBot^®^ parameters such as the ‘scale’ as a measure of the magnitude of the lactation curve, the ‘ramp’ or steepness of the post-parturient rise in milk production, and the ‘decay’ or the rate of late lactation decline were included for analysis. Using the MilkBot^®^ model, milk yield at 60 days of lactation and milk yield at the peak of the lactation curve were calculated^57^. The dry-off date was also included to calculate the length of the dry period.

Sire information was limited to bull identification. For the 210 calves, 90 different sires were recorded. Most of them (59/90) sired only one or two calves. Eight bulls were used on more than five dams, with a maximum of seven calves born out of one sire. Only three bulls were used in more than one herd.

Weather data were obtained from the Royal Observatory of Belgium (Brussels, Belgium) and the Belgian Royal Meteorological Institute (Brussels, Belgium). Weather data included average and maximum temperature, relative humidity, hours of daylight, and hours of total sunlight for each day between 1 October 2016 and 31 December 2018. This timeframe includes weather data starting from the month prior to conception of the first born calf, until one month after birth of the last born calf.

Based on the weather data, a daily temperature-humidity index (THI) was calculated using the relative humidity (RH) and the daily maximum temperature (T), using the following formula: $THI=\mathrm{0,8}*T+RH*\left(T-\mathrm{14,4}\right)+\mathrm{46,4}$^64^. Daily THI’s and median weekly THI’s were calculated. Gestation was divided into trimesters and for each trimester, average THI, number of days with THI greater than 65 and number of days with THI greater than 70 were computed.

### Blood sampling

Whole blood samples were collected, using 10.0 ml BD Vacutainer^®^ tubes (Becton Dickinson, Belliver Industrial Estate, Plymouth PL6 7BP, United Kingdom), with EDTA as an anticoagulant. Blood samples were taken by venepuncture (*Vena jugularis*), within the first 10 days of life and were stored at – 30 °C until further analysis.

### Ethics statement

All experimental procedures were approved by the Ethical Committee (EC) of the Faculty of Veterinary Medicine (Ghent University, Belgium) under the EC number 2017/87. Samples were taken in accordance with the relevant guidelines and regulations and all authors complied with the ARRIVE guidelines^65^.

### DNA extraction and TL measurement

Telomere length was measured at the Centre for Environmental Sciences, at Hasselt University in Belgium.

Leukocyte DNA was extracted from the whole blood using the QIAamp DNA Mini Kit (Qiagen, Inc., Venlo, the Netherlands). DNA yield and purity were assessed for each sample on a NanoDrop 1000 spectrophotometer (Isogen, Life Sciences, Belgium) and DNA integrity was evaluated with agarose gel-electrophoresis.

Relative average leukocyte TL was measured and assessed in triplicate, as previously described by Martens et al.^9^, by the use of a modified quantitative real-time PCR (qPCR) protocol. Briefly, the telomeric region was amplified with the use of telomere specific primers (telg and telc), and one single-copy gene (beta-globulin) was amplified on a QS5 Fast Real-Time PCR System (Applied Biosystems, Hasselt, Belgium) in a 384-well format. Cycle thresholds after the amplification of the telomere specific region were normalized relative to the cycle thresholds after the amplification of the single-copy gene using the QBase+2 software (Biogazelle, Zwijnaarde, Belgium)^9^.

Relative average leukocyte telomere lengths were expressed as the ratio of telomere copy number to single-copy gene number (T/S), relative to the average T/S ratio of the entire sample set. Reaction efficiency was assessed on each reaction plate (using a 6-point serial dilution of pooled buffy coat DNA) and two inter-run calibrators were used to account for inter-run variability.

Coefficients of variation (CVs) of 0.48%, 0.29% and 5.97% for telomere runs, single-copy gene runs and T/S ratios, respectively, were achieved. The reliability of our assay was evaluated by calculating the intraclass coefficient (ICC) with 95% CI of triplicate measures (T/S ratios). The intra-assay ICC was 0.849 (95% CI 0.812 to 0.879).

### Statistical analysis

All statistical analysis were performed in R 3.6.1^66^.

As TL was not normally distributed, all TL measurements were log_10_ transformed to achieve normal distribution (Shapiro-Wilkinson normality test: W=0.99381, P=0.5338). To assess factors associated with log_10_ transformed TL, linear mixed models were built using the lmer() function of the ‘lme4’ package^67^.

The outcome variable for all models was the log_10_ transformed TL, and herd was included as a random effect. The fixed effects of interest were related to the calf, its dam and the environment. Calf related variables were conception month and season, birth month and season, age at sampling, body weight as well as the different body measurements. The examined dam related variables were dam parity (both as a continuous and categorical variable), dam age, gestation length, age at conception for the primiparous animals, and parturition-to-conception interval, calving interval, dry period length, and MilkBot® parameters of the previous lactation for multiparous animals. Daily THI’s, weekly THI’s, and mean THI per trimester of gestation were investigated as well.

First, univariable associations between the outcome variable and independent factors were examined with statistical significance assessed at P < 0.15. Second, correlation coefficients were calculated between the significant variables to avoid multicollinearity in the next step. A correlation coefficient > 0.60 among two factors led to the selection of one of the two variables for further analysis, based on significance and physiological relevance. The selected fixed effects and their 2-way interactions were combined into a multivariable model, but removed if found non-significant, after which the model was refitted. Modelling was performed using a backward stepwise elimination method with a selection criterion based on the Akaike information criterion (AIC). Statistical significance and tendency were declared at P < 0.05 and 0.05 < P < 0.1, respectively.

First a multivariable model was built for all animals. A second model was built for multiparous dams, since milk yield parameters were only available for calves born out of lactating cows. Coefficients of determination (R^2^ analogs) for both models were calculated using the r.squaredGLMM() function^68^. Regression estimates with their 95% CI are presented as a % difference in TL for each unit increase in explanatory variable. Figures were generated with the library ‘ggplot2’^69^.

## Supplementary Information

Supplementary Information.

## Data Availability

Datasets used in this manuscript are available from the corresponding author on reasonable request.
