# Supplementary material for: Prenatal environment impacts telomere length in newborn dairy heifers
Source: Sci Rep. 2023 Mar 22;13:4672. doi: 10.1038/s41598-023-31943-8 (PMC10033676; doi:10.1038/s41598-023-31943-8)
Supplement: Supplementary file 1 — Supplementary Information. [file 41598_2023_31943_MOESM1_ESM.pdf]

Prenatal environment impacts telomere length in newborn dairy heifers

Maya Meesters[1,*], Mieke Van Eetvelde[1], Dries S. Martens[2], Tim S. Nawrot[2,3], Manon Dewulf[1], Jan Govaere[1], Geert Opsomer[1]

[1]Department of Internal Medicine, Reproduction and Population Medicine, Faculty of Veterinary Medicine, Ghent University, Merelbeke, Belgium

[2]Centre for Environmental Sciences, Hasselt University, Diepenbeek, Belgium

[3]Research Unit Environment and Health, Department of Public Health & Primary Care, Leuven University, Leuven, Belgium

*Corresponding author:

Maya Meesters

Department of Internal Medicine, Reproduction and Population Medicine, Faculty of Veterinary Medicine, Ghent University, Merelbeke, Belgium

Maya.Meesters@ugent.be

Supplementary file S1: Calf and dam characteristics of the studied population within each herd

Table 1: Calf and dam characteristics of the studied population within each herd

**Characteristic**	**Mean ± SD or n (%)**				**P-value***
	**Herd I**	**Herd II**	**Herd III**	**Herd IV**	
Calves	46	82	44	38	
Birth season					0.009
Spring	2 (4.3)	13 (15.9)	0 (0.0)	5 (13.2)	
Summer	14 (30.4)[a]	23 (28.0)[a]	24 (54.6)[b]	7 (18.4)[a]	
Fall	23 (50.0)	35 (42.7)	17 (38.6)	19 (50.0)	
Winter	7 (15.2)	11 (13.4)	3 (6.8)	7 (18.4)	
Gestation length (days)	281 ± 4.2[a]	276 ± 3.9[b]	275 ± 4.4[ab]	280 ± 4.0[ab]	0.012
Age at sampling (days)	5 ± 2.5[a]	4 ± 2.2[b]	4 ± 1.8[ab]	4 ± 2.2[ab]	0.040
Body weight at sampling (kg)	41.3 ± 4.34	40.8 ± 4.87	40.0 ± 5.38	39.4 ± 3.84	0.217
Heart girth at sampling (cm)	79.8 ± 3.11	79.1 ± 3.21	78.9 ± 3.75	78.8 ± 2.56	0.436
Withers height at sampling (cm)	75.7 ± 1.85	76.4 ± 3.18	75.8 ± 3.34	76.4 ± 2.78	0.495
Diagonal length at sampling (cm)	72.7 ± 2.76	72.6 ± 3.23	72.2 ± 4.10	71.9 ± 2.92	0.647
Dams					
Parity					0.31
Primiparous dams	14 (30.4)	39 (47.6)	19 (43.2)	16 (42.1)	
Multiparous dams	32 (69.6)	43 (52.5)	25 (56.8)	22 (57.9)	
Age at parturition (months)					
Primiparous	24.4 ± 1.9[ab]	22.2 ±1.3[c]	23.6 ±[a] 1.1	25.2 ± 1.5[b]	<0.001
Multiparous	55.1 ± 18.3	47.3 ± 15.2	46.3 ± 10.9	52.4 ± 20.8	0.120
Calving interval (days)	393 ± 72.8	380 ± 52.4	356 ± 23.5	385 ± 77.2	0.119
Dry period (days)	47 ± 18.1	43 ± 6.7	39 ± 5.9	45 ± 12.9	0.090
Milk yield during gestation (kg)	**5803** ± 1323.5[a]	**8292** ± 1437.6[b]	**6937** ± 1138.3[c]	**6863** ± 1198.1[c]	< 0.001
During first trimester	**2602** ± 559.9[a]	**3539** ± 658.6[b]	**3087** ± 526.0[c]	**2950** ± 501.8[ac]	<0.001
During second trimester	**2105** ± 423.8[a]	**3071** ± 530.6[b]	**2513** ± 412.7[c]	**2521** ± 424.3[c]	<0.001
During third trimester	**1097** ± 463.3[a]	**1681** ± 400.2[b]	**1337** ± 298.9[ac]	**1393** ± 375.9[c]	<0.001

*Significant differences between herds were estimated using ANOVA (for continuous variables) or Chi-square analysis (for categorical variables). Different superscripts indicate significant differences between herds (P<0.05)

Supplementary file S2: Distributions of the untransformed an log_{10}-transformed TL

The calves' untransformed TL was on average 1.01 ± 0.17, ranging from 0.66 to 1.66. The median (P50) was 0.99 and the 25th (P25) and 75th (P75) percentile were 0.90 and 1.12, respectively (Figure 1: Distributions of the untransformed TL).

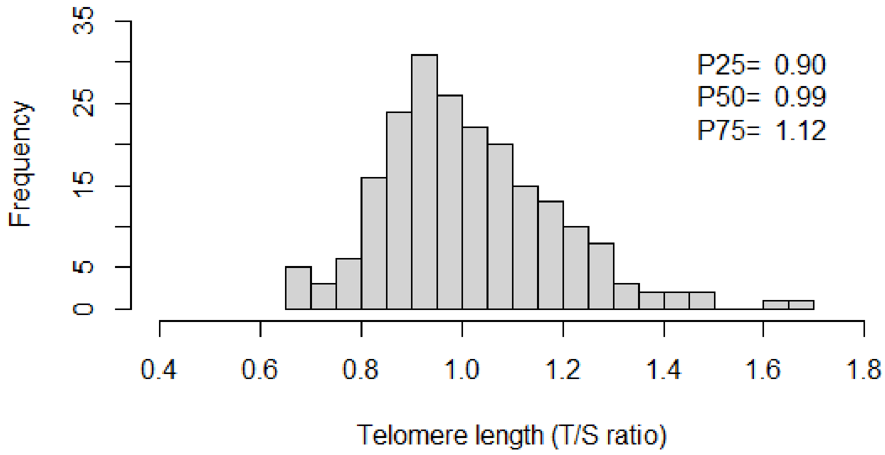

Figure 1: Distributions of the untransformed TL

The log_{10}-transformed TL of the calves was on average 0.00 ± 0.07, ranging from -0.18 to 0.22. The 25th percentile, median and 75th percentile were -0.05, 0.00 and 0.05, respectively (Figure 2: Distributions of the log10-transformed TL).

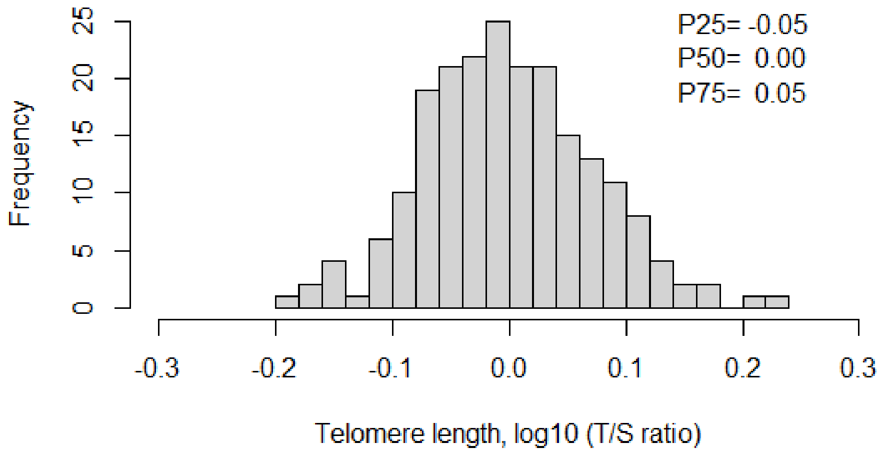

Figure 2: Distributions of the log10-transformed TL

Supplementary file 3: detailed weekly THI's between week 20 and week 39

Table 2: Results of the univariable model showing the association between weekly THI's and the log_{10} transformed TL

Variables	Categories	Estimates[a]	P-value
**Weekly THI**			
	Week 20	-0.098	0.361
	Week 21	-0.143	0.195
	Week 22	-0.178	0.113
	Week 23	-0.156	0.187
	Week 24	-0.093	0.415
	Week 25	-0.114	0.020
	Week 26	-0.174	0.001
	Week 27	-0.161	0.003
	Week 28	-0.161	0.002
	Week 29	-0.168	<0.001
	Week 30	-0.137	0.004
	Week 31	-0.161	<0.001
	Week 32	-0.584	<0.001
	Week 33	-0.155	0.002
	Week 34	-0.145	0.003
	Week 35	-0.125	0.006
	Week 36	-0.115	0.018
	Week 37	-0.074	0.104
	Week 38	-0.077	0.071
	Week 39	-0.036	0.403

[a]Estimates presented as a % difference in TL for a 1-unit increase in the explanatory variable
